# Partial response to trastuzumab deruxtecan (DS8201) following progression in HER2-amplified breast cancer with pulmonary metastases managed with disitamab vedotin (RC48): a comprehensive case report and literature review

**DOI:** 10.3389/fonc.2024.1338661

**Published:** 2024-06-17

**Authors:** Yanfang Lan, Jiahui Zhao, Fangrui Zhao, Juanjuan Li, Xiangpan Li

**Affiliations:** ^1^ Department of Oncology, Renmin Hospital of Wuhan University, Wuhan, Hubei, China; ^2^ Department of Breast and Thyroid Surgery, Renmin Hospital of Wuhan University, Wuhan, Hubei, China

**Keywords:** antibody-drug conjugate, trastuzumab deruxtecan, disitamab vedotin, tislelizumab, Her2-amplified breast cancer

## Abstract

Breast cancer remains one of the predominant malignancies worldwide. In the context of inoperable advanced or metastatic human epidermal growth factor receptor 2 (HER2)-positive breast cancer, systemic management primarily relies on HER2-targeting monoclonal antibodies. With the successful development of anti-HER2 antibody-drug conjugates (ADCs), these agents have been increasingly integrated into therapeutic regimens for metastatic breast cancer. Here, we present the case of a 42-year-old female patient with HER2-positive pulmonary metastatic breast cancer who underwent an extensive treatment protocol. This protocol included chemotherapy, radiation therapy, hormonal therapy, surgical intervention on the breast, and anti-HER2 therapies. The anti-HER2 therapies involved both singular and dual targeting strategies using trastuzumab and the ADC disitamab vedotin (RC48) over an 8-year period. After experiencing disease progression following HER2-targeted therapy with RC48, the patient achieved noticeable partial remission through a therapeutic regimen that combined trastuzumab deruxtecan (DS8201) and tislelizumab. The data suggest a promising role for DS8201 in managing advanced stages of HER2-amplified metastatic breast cancer, especially in cases that demonstrate progression after initial HER2-directed therapies using ADCs. Furthermore, its combination with anti-PD-1 agents enhances therapeutic efficacy by augmenting the anti-tumoral immune response.

## Introduction

Breast cancer remains a predominant malignancy worldwide. Incorporating both sexes, it accounts for 11.6% of all cancer cases, second only to lung cancer. Notably, among women, it is the most frequently diagnosed cancer (1). Human epidermal growth factor receptor 2 (HER2)-positive breast cancers make up 25–30% of all breast cancer cases, often indicating a worse prognosis compared to luminal subtype (2–4). Due to tumor heterogeneous, HER2 positive breast cancer present various treatment sensitivities and different survival outcomes. A correlation exists between the development of distant metastases and increased mortality rates in breast cancer patients (5). Common metastatic sites include the bone, liver, lung, and brain across all breast cancer subtypes. Advanced systemic therapy for inoperable or metastatic HER2-positive breast cancer primarily relies on HER2-targeting monoclonal antibodies. Currently, the standard of care in initial and second-line settings involves pertuzumab and trastuzumab in conjunction with chemotherapy, preferably a taxane, trastuzumab emtansine (T-DM1) and trastuzumab deruxtecan (DS8201) (6). Subsequent lines of therapy may consider alternative HER2-targeted combinations, including options such as or tyrosine kinase inhibitors (TKIs) like tucatinib and neratinib (7).

Antibody-drug conjugates (ADCs) consist of tumor antigen-specific monoclonal antibodies bound to potent cytotoxic agents via stable, cleavable, or non-cleavable chemical linkers. Due to successful advancements in ADC pharmaceuticals, these agents have been progressively incorporated into treatment protocols for various diseases. Disitamab vedotin (RC48) is an example of an anti-HER2 ADC; it combines hertuzumab (a novel anti-HER2 mAB) with monomethyl auristatin E (MMAE) through a cleavable linker (8). A consolidated analysis of a phase I dose-escalation study (NCT02881138) and a parallel open-label phase Ib trial (NCT03052634) showed that HER2-positive breast cancer patients achieved an objective response rate (ORR) of 31.4%, along with a median progression-free survival (PFS) of 5.8 months (9, 10). DS-8201 is another ADC, featuring an anti-HER2 antibody linked to a cytotoxic topoisomerase I inhibitor through a cleavable tetrapeptide-based linker. As highlighted in the global phase 2 study DESTINY-Breast01, DS-8201 exhibited significant clinical efficacy in HER2-positive metastatic breast cancer patients who had received extensive prior therapies, including treatment with pertuzumab or T-DM1 (11). Despite the encouraging prospects of both RC48 and DS8201 in breast cancer management, existing literature lacks reports on the effectiveness of DS8201 following a failed RC48 regimen.

**Figure 1:**
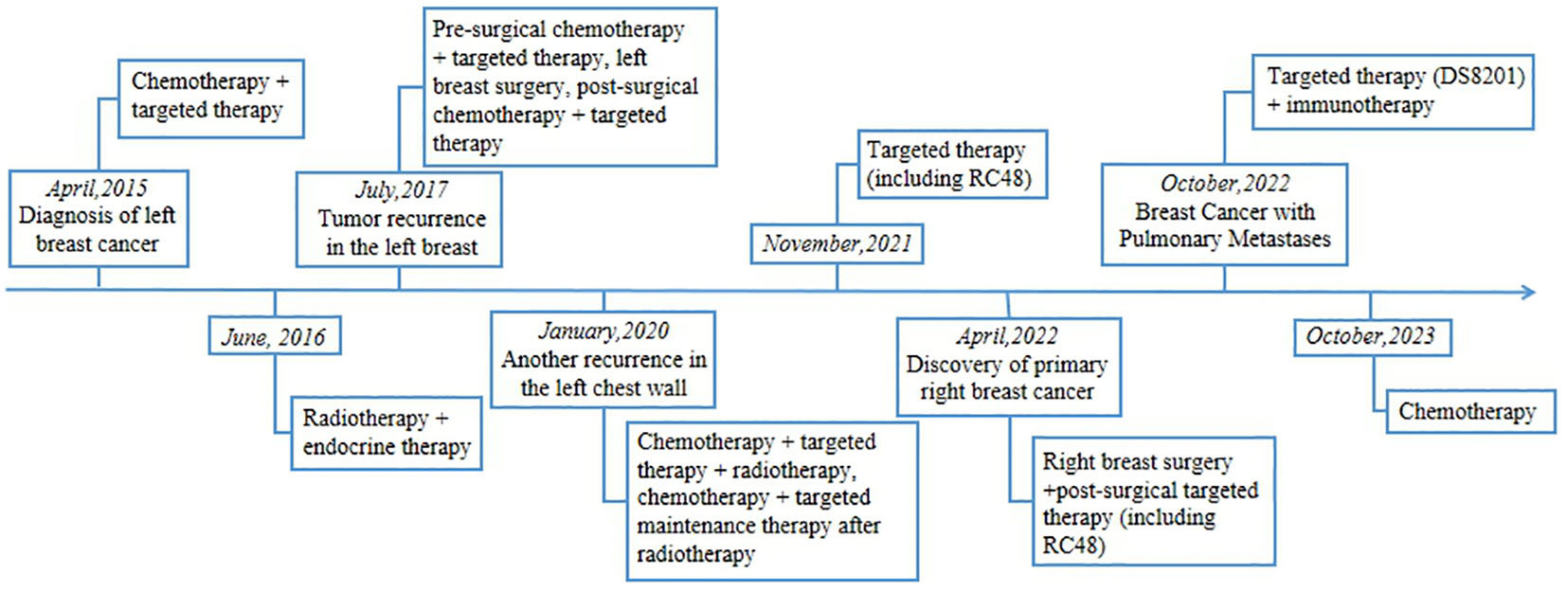
Patient’s Treatment Timeline.

In this report, we describe a patient with HER2-positive breast cancer, characterized by pulmonary metastases, who exhibited resistance to RC48 treatment after undergoing a range of therapeutic interventions. These interventions included chemotherapy, radiation therapy, targeted therapies, and surgical intervention on the breast. Remarkably, the patient responded positively to a treatment regimen that included DS8201 and tislelizumab (**Figure 1**).

## Case presentation

**Figure 2:**
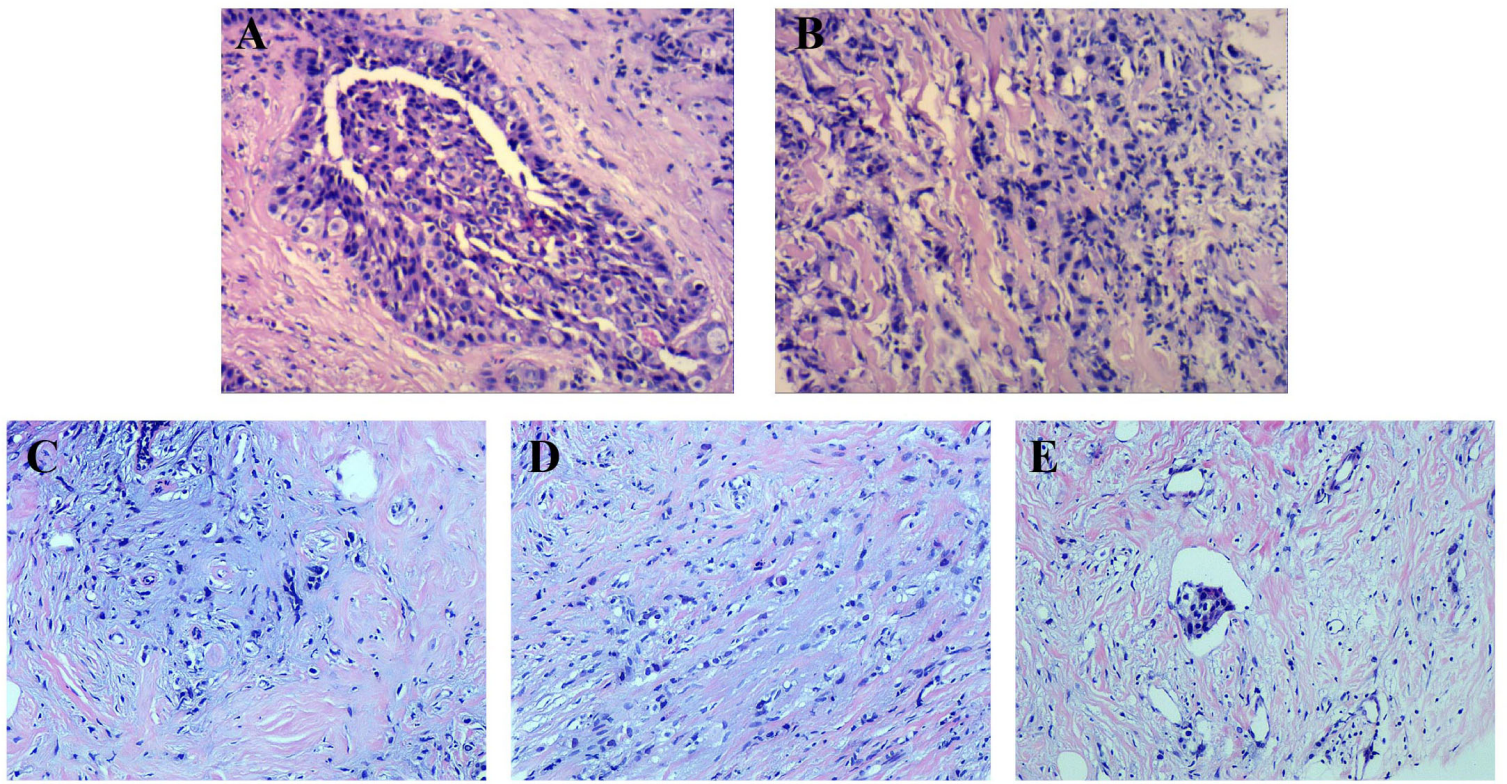
Histopathologic Examinations of the Primary Tumor Focus in the Left Breast and the Right Breast Before Operation. **(A, B)** HE-stained images of the primary tumor focus (100×). **(C–E)** HE-stained images of the tumor focus on the right breast (40×).

In April 2015, a 42-year-old woman who has no family history of breast, ovarian or other cancers noticed bilateral breast asymmetry, minor enlargement of the left breast, and palpable enlargement of lymph nodes in the medial left breast. Subsequent pathological examination of a left breast aspirate revealed ER (+20%), PR (-), HER2 (3+), Ki-67 (+80%). The clinical diagnosis was grade III invasive ductal carcinoma of the left breast with axillary lymph node metastasis [TNM stage: T3N2acM0 (i+)] (**Figures 2A, B**). Following the diagnosis, the patient began a treatment regimen consisting of 4 cycles of EC regimen (epirubicin 100 mg/m^2^, Q21d and cyclophosphamide 600 mg/m^2^, Q21d), followed by 4 cycles of TH regimen (docetaxel 100 mg/m^2^, Q21d and trastuzumab, 8mg/kg on day1, then 6mg/kg Q21d), along with one year of trastuzumab-targeted therapy (6 mg/kg, Q21d). In June 2016, after declining a recommendation for radical mastectomy, she received radiation therapy targeting the left supraclavicular lymphatic drainage area and axillary lymph nodes (CTV: 25F/50Gy), as well as the left breast (CTV: 25F/50Gy). She also initiated endocrine therapy with a combination of goserelin (Zoladex 3.6mg, Q28d), a luteinizing hormone-releasing hormone analog, and anastrozole (1mg, QD), an aromatase inhibitor.

In July 2017, the patient experienced tumor recurrence in the left breast, leading to the initiation of 6 cycles of TH regimen (docetaxel 100 mg/m^2^, Q21d and trastuzumab 8mg/kg on day1, then 6mg/kg Q21d), along with another year of trastuzumab-targeted therapy (6 mg/kg, Q21d). In November 2017, she underwent a total left mastectomy with axillary lymph node dissection at Renmin Hospital of Wuhan University. Postoperative assessments, which included immunohistochemistry, revealed the following markers: E-Cad (+), ER (-), HER2 (3+), Ki-67 (+10%), P120 (+), P63 (-), and PR (-). After the surgery, she stopped the endocrine therapy and received a chemotherapy regimen combined with targeted therapy (capecitabine 1000 mg/m^2^, Bid, two consecutive weeks followed by a one-week break and pyrotinib 400mg, QD).

The onset of 2020 brought another recurrence in the left chest wall. Consequently, her treatment strategy was revised to include chemotherapy combined with targeted therapy, specifically albumin paclitaxel (260 mg/m^2^, Q21d), along with trastuzumab (8mg/kg on day1, then 6mg/kg Q21d) and pertuzumab (840mg on day1, then 420mg Q21d), for a total of six cycles. She then underwent radiation therapy targeting the left chest wall (CTV: 2Gy/33F/66Gy) and continued maintenance treatment with capecitabine (1000 mg/m^2^, Bid, two consecutive weeks followed by a one-week break) and pyrotinib (400mg, QD) post-radiotherapy.

In November 2021, the patient switched to an RC48 regimen (2.5mg/kg, Q14d) following a right breast aspiration biopsy that revealed tumor cells (**Figures 2C–E**). After six cycles, her treatment regimen was modified to RC48 (2.5mg/kg, Q14d) and apatinib (850mg, QD), which is a tyrosine kinase inhibitor that selectively inhibits the vascular endothelial growth factor receptor-2. On April 1, 2022, she underwent a mastectomy along with axillary lymph node dissection for right breast cancer. Histopathology revealed primary right breast cancer with axillary lymph node involvement (11/34), and immunohistochemistry showed the following markers: invasive carcinoma AR (+70%), CK5/6 (-), E-Cadherin (+), ER (-), HER2 (3+), Ki-67 (+60%), P120 (membrane+), P63 (-), PR (-). After the surgery, she began a treatment regimen comprising RC48 (2.5mg/kg, Q14d) and pertuzumab (420mg, Q21d).

**Figure 3:**
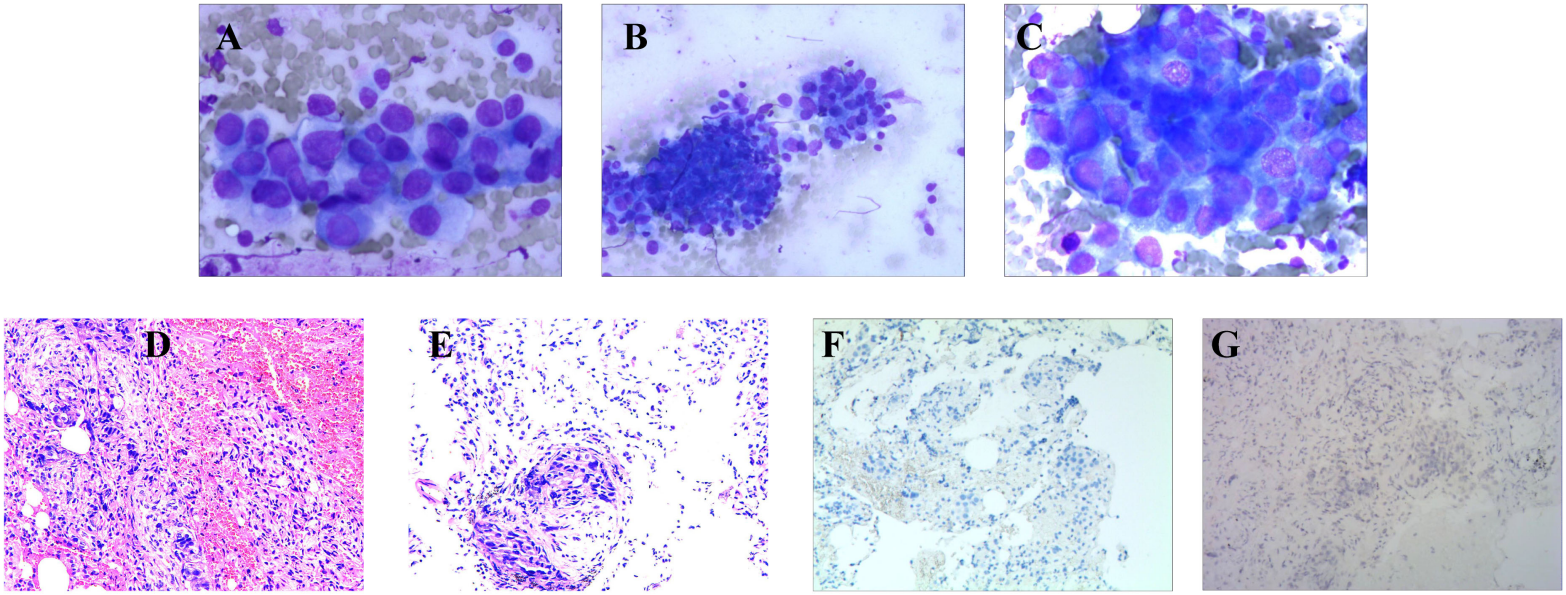
Brush Cytology, Histopathologic, and Immunohistochemical Examinations of Biopsy Tissue from Pulmonary Metastasis. **(A–C)** Light microscope images of brush cytology; cancer cells are visible (**A, C**: 200×; **B**:100). **(D, E)** HE-stained images of biopsy tissue from the pulmonary metastasis focus (40×). **(F, G)** PD-L1: Negative (**F**: 40×; **G**: 100×).

Concurrent with her earlier treatments, the patient began experiencing coughing symptoms in December 2021. Despite receiving anti-infective treatment, the cough persisted. In September 2022, her respiratory symptoms worsened, leading to resting dyspnea. A subsequent ciliary bronchoscopy at our facility confirmed the presence of cancer cells (**Figures 3A–C**). A bronchial specimen biopsy was then conducted, and pathological results showed metastatic invasive carcinoma of the breast, thus clinically indicating breast cancer metastasis (**Figures 3D, E**). Immunohistochemistry results showed negative expression for programmed cell death ligand 1 (PD-L1), with a Combined Positive Score (CPS) of 0 (**Figures 3F, G**). CA125 level measured on October 10, 2022, was 96.2 U/ml. A chest CT scan from the same date showed carcinomatous lymphangitis in a lung lesion. Consequently, the treatment swith to the combination of DS8201 (200mg, q21d) and anti-PD-1 immunotherapy (tislelizumab 200mg, q21d).

**Figure 4:**
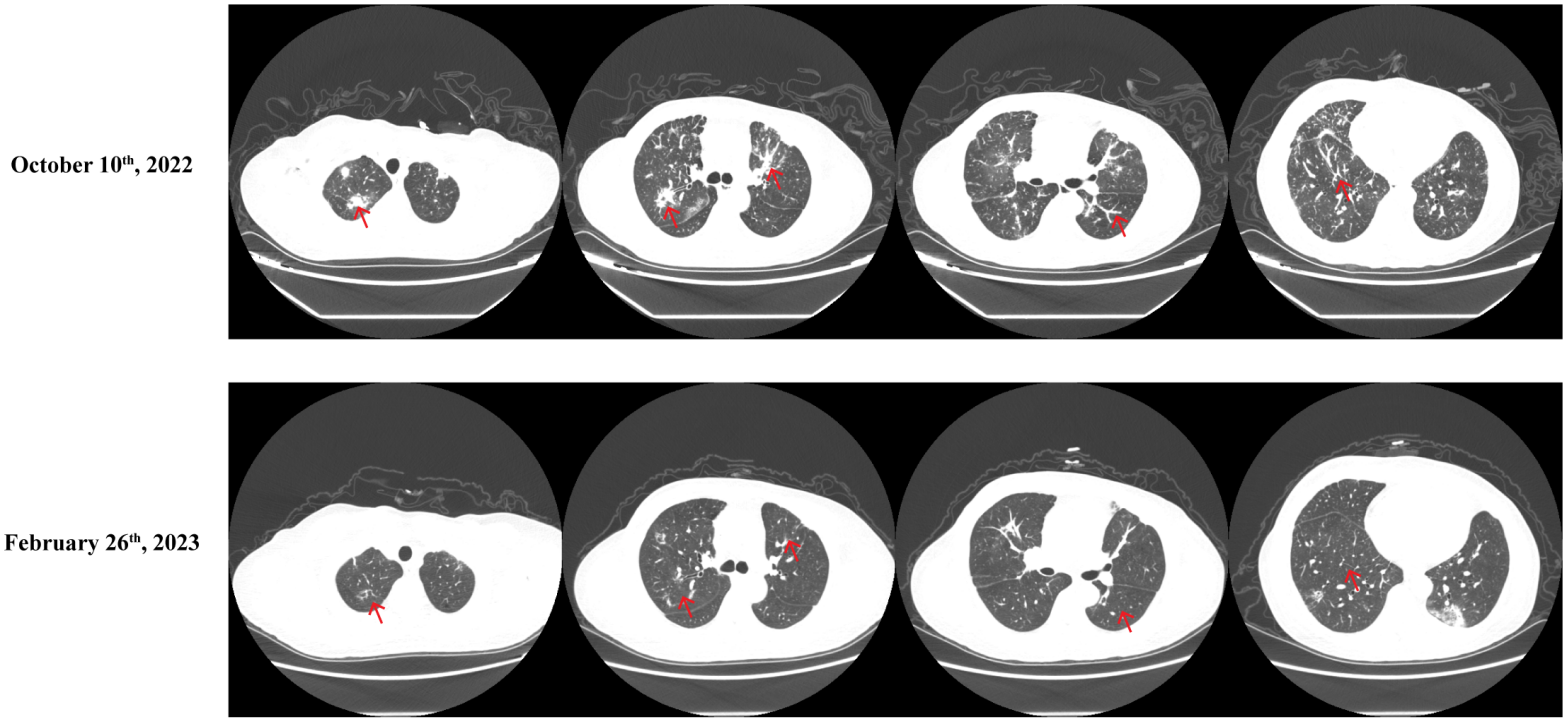
Comparative Chest CT Scans Conducted Before and After the Fifth DS8201 and Tislelizumab Treatment Regimen Cycle. Before: October 10, 2022, After: February 26, 2023.

After two cycles of that treatment, a significant alleviation of the symptoms of resting dyspnea was observed. After five cycles, a comparative analysis with pre-treatment CT scans revealed a notable reduction in the size of multiple lung nodules and nodular foci and improved carcinomatous lymphangitis (**Figure 4**). On February 24, 2023, the level of CA125 was 32.9 U/ml, indicating a decrease compared to the previous measurement. The treatment efficacy was evaluated as a partial response (PR). The following adverse effects were observed during treatment and markedly relieved by symptomatic treatments: nausea, fatigue, vomiting, alopecia, constipation, and decreased appetite. No severe or life-threatening adverse events were reported. Unfortunately, after 12 cycles, the response was evaluated as PD. In October 2023, she began a chemotherapy regimen of gemcitabine (1000mg/m^2^, days 1 and 8 of a 21-day cycle) plus nedaplatin (80mg/m^2^, Q21d) and underwent genetic testing, which revealed the detection of two somatic mutations associated with targeted drugs, HER2 (amplification) and PIK3CA (exon 21, c.3140A > G, with alteration of protein p.H1047R), but no BRCA1 or BRCA 2 mutations. However, a deterioration in physical status and a severe lung infection were noted after 3 cycles. Regrettably, the patient passed away in late January 2024.

## Discussion

This report outlines the case of a young patient battling advanced pulmonary metastases stemming from HER2-amplified breast cancer. She has undergone a myriad of treatments for recurrent conditions throughout her illness, including chemotherapy, radiotherapy, targeted therapy, and surgery. Notably, she demonstrated a significant response to a treatment regimen that included DS8201 and tislelizumab, despite disease progression under RC48 and other therapies. To our knowledge, this represents a unique and unprecedented case in the literature on breast cancer.

Prior studies have revealed that the prognosis of breast cancer correlates with the expression levels of estrogen receptor (ER) and progesterone receptor (PR) (12). And just as several earlier studies have reported differences in ER and PR expression between primary tumors and recurrent or metastatic tumors, we noticed the loss of ER expression after recurrence in the left breast in this case (13, 14). Possible reasons for this phenomenon include technical problems with poor reproducibility of immunohistochemistry, tumor heterogeneity (15, 16). In addition, the patient had a PI3KCA mutation. Previous studies have indicated that the PI3K/AKT/mTOR pathway is associated with the maintenance of endocrine resistance (17). The loss of ER expression along side the PIK3CA mutation were likely significant factors that could have contributed to endocrine treatment resistance. And the data from a retrospective study demonstrated that in patients with early-stage breast cancer, after adjusting treatment according to receptor expression in recurrent/metastatic lesions, the majority of patients were maintained progression-free during the follow-up period (18).

HER2-positive tumors are intrinsically linked to poorer survival outcomes compared to cases characterized by estrogen receptor (ER)-positive, HER2-negative breast cancer. However, the past three decades have seen relentless research and development of anti-HER2 agents, leading to significantly improved prognoses for patients diagnosed with both early and advanced stages of HER2-positive breast cancer. Alternative therapeutic agents for HER2-positive breast cancer range from HER2-targeted monoclonal antibodies (such as trastuzumab and pertuzumab), HER2-targeted ADCs (like T-DM1 and DS8201), and small molecule tyrosine kinase inhibitors (including lapatinib, neratinib, pyrotinib and tucatinib). Regrettably, the primary guidelines, which recommend dual blockade with trastuzumab and pertuzumab as a first-line treatment followed by T-DM1 as a second-line treatment, have remained unchanged since 2012. Nevertheless, the landscape underwent a significant transformation after 2019, marked by an influx of clinical trials and subsequent approval of three novel agents: DS-8201, tucatinib, and neratinib. This development signals a promising shift in the therapeutic landscape (19). DS-8201, classified as an ADC, is particularly noteworthy.

ADCs constitute a complex fusion of monoclonal antibodies specific to tumor antigens, combined with stable chemical linkers—either cleavable or non-cleavable—and highly potent cytotoxic agents. The ADC-antigen complex is internalized upon binding to the target, primarily through clathrin-mediated endocytosis (20). This triggers a cascade of intracellular events, starting with forming an early endosome, which matures into a late endosome before undergoing lysosomal fusion. The fate of the ADC depends on the type of linker it possesses: cleavable linkers are subject to mechanisms such as hydrolysis, enzymatic cleavage by proteases, or reductive cleavage of disulfide bonds, primarily within the cytoplasm, thereby bypassing lysosomal transport. Conversely, ADCs with non-cleavable linkers require complete proteolytic degradation within the lysosome.

After its intracellular release, the cytotoxic component induces cell death through mechanisms like DNA intercalation or inhibition of microtubule polymerization. This triggers a series of events within the cellular environment, potentially including the bystander killing of adjacent tumor cells and stromal tissue, which may have absorbed the drug through diffusion, depending on the hydrophobic properties of the cytotoxic payload (21). Moreover, ADCs play a crucial role in activating complement systems and facilitating the influx of immune effector cells at the tumor site, using various strategies like complement-dependent cytotoxicity (CDC), antibody-dependent cellular cytotoxicity (ADCC), or antibody-dependent cellular phagocytosis (ADCP) (22). For example, T-DM1 has shown efficacy in increasing the population of tumor-infiltrating lymphocytes within primary human breast cancers and promoting effector T-cell infiltration in murine breast tumors (23).

As of March 2023, the FDA has globally approved 15 ADC medications. Notably, T-DM1 and DS8201 have received FDA approval for managing HER2-positive breast cancer, as supported by the results of the EMILIA, KATHERINE, and DESTINY-Breast01 clinical trials (11, 24, 25). Specifically, T-DM1 is approved for treating previously treated HER2-positive metastatic breast cancer. At the same time, DS8201 received expedited FDA approval for patients with unresectable or metastatic HER2-positive breast cancer who have undergone at least two anti-HER2-based treatment regimens in a metastatic setting, aligning closely with the current case under discussion. Additionally, DS8201 is approved for treating adult patients with locally advanced or metastatic HER2-positive gastric or gastroesophageal junction cancer (GC/GEJC), provided they have received prior trastuzumab-based therapy. The striking response of a patient with metastatic HER2-amplified and L755S-mutated breast cancer to T-DM1 and DS8201, after showing resistance to other HER2-targeted drugs, exemplifies the specific anticancer potential of anti-HER2 ADCs (26).

Numerous clinical studies have demonstrated the efficacy of RC48 in treating patients with either locally advanced or metastatic HER2-overexpressing gastric cancer, including adenocarcinoma of the gastroesophageal junction, following a minimum of two systemic chemotherapy courses (27–29). It is also effective for those with advanced or metastatic uroepithelial cancer who have previously received platinum-containing chemotherapy and have shown HER2 overexpression of either 2+ or 3+ on immunohistochemistry (29, 30). In June 2021, China’s Center for Drug Evaluation designated RC48 as a breakthrough therapy for HER2-positive individuals with metastatic breast cancer (MBC) with advanced liver metastases who had undergone treatment with trastuzumab and paclitaxel. Subgroup analyses from two studies published by the American Society of Clinical Oncology outline the benefits of RC48 treatment, regardless of the presence of HER2 gene mutations or fusions (31). In one specific case, a patient with stage IV (cT4N3M1) hormone receptor (HR)-positive and HER2-positive invasive ductal carcinoma, who presented with systemic metastases including the brain, underwent 26 cycles of initial anti-HER2 targeted therapy along with chemotherapy. After experiencing disease progression, the patient received four cycles of second-line therapy (trastuzumab + piritinib + capecitabine), which unfortunately led to further disease progression. The patient then received 12 cycles of RC48 as the third-line therapy, which resulted in significant benefit without severe adverse effects and extended overall survival beyond three years (32). Therefore, advanced breast cancer patients stand to benefit from RC48 therapy. Ongoing clinical trials are further investigating the role of RC48 in the therapeutic landscape of breast cancer. As a result, it appears reasonable to hypothesize that RC48 could play a significant role in achieving remission in patients with HER2-positive breast cancer, even though its current indication does not include breast cancer.

The mechanisms underlying resistance to anti-HER2 ADCs are diverse, as elucidated through studies focusing on T-DM1 and DS8201. Firstly, resistance may arise from reduced HER2 levels or structural changes in the receptor (33, 34). Secondly, altered HER2 internalization processes can also contribute to resistance. Specifically, the endomorphin A2 protein aids in internalizing complexes formed post-HER2 binding by anti-HER2 ADCs, ultimately leading to endosome formation. Notably, silencing this protein in HER2-positive cells has inhibited HER2 internalization, reducing responsiveness to agents like trastuzumab and T-DM1 (35). Additionally, the ubiquitination and subsequent transport of HER2 to lysosomes could be modified due to its relationship with the chaperone protein HSP90. The combined action of 17-AAG-mediated HSP90 inhibition and trastuzumab enhances HER2 endocytosis into lysosomes, promoting further degradation within these organelles (36). Moreover, an increased recycling rate of endosomes containing HER2 back to the plasma membrane could limit the delivery of T-DM1 to the lysosome, thus restricting its intracellular release. This could be attributed to the rapid recycling rate of HER2 observed following trastuzumab binding (37). Thirdly, resistance might occur due to changes in lysosomal functions (38). The efficient transport of anti-HER2 ADCs to lysosomes and their subsequent processing by lysosomal enzymes are crucial for liberating the attached payloads. Lysosomes are intrinsically acidic and contain proteolytic capabilities regulated by the vacuolar proton pump H+-ATPase (V-ATPase), crucial in maintaining lysosomal pH. Fourthly, resistance could emerge from increased expression and functionality of plasma membrane drug efflux pumps, a mechanism widely studied in the realm of chemotherapy resistance. Regarding T-DM1, it has been found that specific ABC family pumps can eject the compound Lys-MCC-DM1 into the extracellular environment, thereby preventing its interaction with tubulin (39). Furthermore, attention should be given to protein modifications integral to signaling pathways. Variations in the expression of cyclin B1, polo-like kinase 1 (PLK1), and PTEN, among others, have been suggested to induce drug resistance to T-DM1 (40–42). Concurrently, SLX4 loss-of-function mutations have been implicated in resistance to DS8201 (43).

In this case, the patient resisted RC48 but responded positively to DS8201 with a tislelizumab regimen. Approximately 60% of HER2-negative metastatic breast cancers display low levels of HER2, as indicated by an immunohistochemical (IHC) score of either 1+ or 2+, along with negative *in situ* hybridization (ISH) results (44, 45). Unfortunately, current HER2-targeted therapies have not improved clinical outcomes for patients with this subtype, leaving them with limited targeted therapy options following the progression of primary treatment and mainly relegating them to single-agent palliative chemotherapy (46).

First-generation ADCs like T-DM1 use non-cleavable linkers to attach the cytotoxic payload to the antibody, thereby preventing its release into the extracellular space. This approach is highly effective against cells with elevated HER2 expression but has limited efficacy against those with low to moderate HER2 levels. Conversely, second-generation ADCs, including DS8201 and RC48, employ cleavable linkers, allowing for partial payload release into the extracellular space and thereby affecting cells that do not overexpress HER2 (47). Clinical trials support this notion; the DESTINY-Breast04 and Daisy studies showed favorable outcomes using DS8201 in treating patients with low HER2-expressing breast cancer and even in those with HER2–0 breast cancer (47, 48). However, T-DM1’s efficacy is constrained in these low-HER2 cases (33, 49).

A distinct advantage of RC48-ADC is its elevated cytotoxicity at low concentrations. The valine-citrulline (VC) linker used in RC48 is stable and undergoes cleavage by histone proteases only upon endocytosis into lysosomes, subsequently liberating the payload to destroy target cancer cells (50). Additionally, the HER2 antibodies in T-DM1 and DS8201 are derivatives of trastuzumab, whereas RC48 employs hertuzumab. Optimized for screening, hertuzumab shows a higher affinity for HER2 targets than trastuzumab, potentially making it more effective against cancers with low or fluctuating HER2 expression levels. In comparison to other HER2-ADC medications, RC-48 demonstrates superior endocytosis, irrespective of V-ATPase activity, and lacks lysosomal resistance (8).

In light of the available evidence, we hypothesize that the patient’s observed resistance to RC-48 primarily stems from increased expression and activity of plasma membrane drug efflux pumps and changes in other genetic and protein components. However, we cannot entirely rule out other potential mechanisms of resistance.

In a similar case, a 67-year-old male with HER2-positive metastatic parotid gland carcinoma was documented. The patient experienced disease progression following a parotidectomy, complemented by adjuvant cisplatin-based chemoradiation, neratinib, and T-DM1. Upon progression on this last HER2-targeted therapy, he showed a complete response to DS8201, which has been sustained for the past seven months (51). Regardless of prior treatment with either RC48 or T-DM1, patients in both cases responded favorably to DS8201. This is consistent with findings from the DESTINY-Breast01 clinical trial, where DS8201 exhibited durable anti-tumor activity in patients with HER2-positive metastatic breast cancer who were previously treated with ADCs. Therefore, considering DS8201 as a subsequent line of therapy for such individuals may be highly beneficial.

A recent case report reported that combined therapy with the RC48 and zibelizumab (a PD-1 inhibitor) achieved successful control of recurrent HER2-positive breast cancer resistant to trastuzumab (52). Moreover, according to the results of former studies, regardless of the PD-L1 expression status, the application of PD-1 monotherapy or the combination of PD-1/PD-L1 inhibitors with chemotherapy can benefit the patients (53, 54). Alongside DS8201, our patient underwent empirical treatment with tislelizumab, a humanized IgG4 anti-PD-1 monoclonal antibody, despite PD-L1 negativity in bronchial specimen biopsy results. Current research highlights that ADCs can enhance anti-tumor immune responses, thus improving the effectiveness of combination therapies. For example, Iwata et al. demonstrated that the combined use of DS8201 and anti-PD-1 antibodies exceeded the efficacy of either treatment alone, possibly due to enhanced T-cell activity and upregulated PD-L1 expression facilitated by DS8201 (55). Similarly, Müller et al. noted the superior efficacy of combining T-DM1 with anti-CTLA-4/anti-PD-1 therapy over monotherapies, attributing this to the potentiation of both innate and adaptive immune responses (56). Studies have also shown that U3–1402, an ADC targeting HER3, amplifies functionalities and infiltration of innate and adaptive immune cells, thereby sensitizing tumors to immunotherapies. Furthermore, preclinical mouse model studies indicated that ADCs could upregulate PD-1 in CD8 T cells and PD-L1 in tumor cells/tumor-associated macrophages, along with increasing tumor-infiltrating lymphocytes, compared to drug controls (23). Therefore, combining ADCs with immunotherapy may inhibit upregulated immunosuppressive pathways, further enhancing tumor control.

In this case, the patient was switched to another ADC drug DS8201 treatment after RC48 resistance, and the two were connected with different cytotoxic agents to avoid cross-resistance. In addition, combined with tislelizumab immunotherapy increased the anti-tumor efficacy to the patient. However, we need to consider that patients may have a decrease in drug effectiveness and an increase in toxic side effects after multiline drug administration. Further studies should be conducted to explore the efficacy and safety of treatment with an alternative ADC drug after ADC drug resistance.

## Conclusion

In conclusion, this case highlights a remarkable response to a DS8201 and tislelizumab regimen in the context of advanced HER2-positive lung metastasis originating from breast cancer previously treated with RC48. The findings offer preliminary evidence underscoring the potential role of DS8201 in managing advanced HER2-amplified lung metastases from breast cancer, particularly in cases that have progressed after initial HER2-targeted therapies. Moreover, combining DS8201 with anti-PD-1 agents could further boost the tumor immune response, enhancing therapeutic efficacy.

## Data availability statement

The original contributions presented in the study are included in the article/supplementary material. Further inquiries can be directed to the corresponding authors.

## Ethics statement

The studies involving humans were approved by the Institutional Ethics Committee of the Faculty of Medicine at Renmin Hospital of Wuhan University. The studies were conducted in accordance with the local legislation and institutional requirements. The participants provided their written informed consent to participate in this study. Written informed consent was obtained from the individual(s) for the publication of any potentially identifiable images or data included in this article.

## Author contributions

YL: Writing – original draft. JZ: Writing – original draft. FZ: Writing – original draft. JL: Writing – review & editing. XL: Writing – review & editing.
